# Decoupling of muscle‐tendon unit and fascicle velocity contributes to the in vivo stretch‐shortening cycle effect in the male human triceps surae muscle

**DOI:** 10.14814/phy2.70131

**Published:** 2024-12-11

**Authors:** Denis Holzer, Daniel Hahn, Ansgar Schwirtz, Tobias Siebert, Wolfgang Seiberl

**Affiliations:** ^1^ Department of Sport and Health Sciences, Biomechanics in Sports Technical University of Munich Munich Germany; ^2^ Faculty of Sport Science, Human Movement Science Ruhr University Bochum Bochum Germany; ^3^ School of Human Movement and Nutrition Sciences University of Queensland Brisbane Australia; ^4^ Department of Motion and Exercise Science University of Stuttgart Stuttgart Germany; ^5^ Department of Human Sciences, Institute of Sport Science Universität der Bundeswehr München Neubiberg Germany

**Keywords:** gearing, joint moment, locomotion, muscle force enhancement, muscle‐tendon dynamics, ultra sound

## Abstract

During the shortening of stretch‐shortening cycles (SSCs), muscle force output is enhanced compared with pure shortening (SHO), referred to as the SSC‐effect. In general, muscle‐tendon unit (MTU), muscle belly, muscle fascicle, and tendon length changes can be decoupled during contraction, which affects force generation and elastic recoil. We researched whether MTU decoupling contributes to the SSC‐effect. Participants performed electrically stimulated submaximal fixed‐end, SSC, and SHO plantar‐flexions on a dynamometer at two velocities (40, 120°/s) and two ranges of motion (15, 25°). Fascicle and tendon length changes of the gastrocnemius medialis, and ankle joint kinematics were assessed by ultrasound and motion capture, respectively. During SSC shortening, ankle joint torque and work, MTU force and work, and fascicle force were increased by 12%–22% compared with SHO, confirming a SSC‐effect. Further, fascicle length change and velocity during SSCs were significantly reduced compared with SHO condition, and SSC fascicle work was decreased by ~35%. Our results indicate that MTU decoupling leads to a reduction in fascicle shortening amplitude and velocity, thereby increasing the muscle's force capacity while reducing its work output during SSC shortening. MTU decoupling therefore contributes to the SSC‐effect and underlines the limited transferability of joint work measurements to estimated muscle work.

## 1 INTRODUCTION

Stretch‐shortening cycles (SSC) are the most common muscle‐tendon unit (MTU) behavior in sports and daily locomotion. A SSC consists of an MTU lengthening, also called active stretch, which is immediately followed by MTU shortening (Komi, 2000). During the shortening phase of the SSC, muscle force, work, and power are increased by up to 50% compared with pure shortening muscle action (Cavagna et al., 1968), which ultimately leads to an optimized performance and reduced (metabolic) energy consumption of the muscle (Aura & Komi, 1986; Dawson & Taylor, 1973). Throughout this manuscript this increase in force, work, and power is referred to as the SSC‐effect.

Although the SSC‐effect has been demonstrated in a large number of experiments over a wide variety of structural levels (in vitro, in situ, in vivo) (Cavagna et al., 1968; Fukutani et al., 2017; Fukutani & Herzog, 2021; Groeber et al., 2021; Hahn & Riedel, 2018; Komi, 2000; Seiberl, Power, Herzog, & Hahn, 2015; Tomalka et al., 2020; van Schenau et al., 1997), as well as in models and simulations (Bobbert et al., 1996; Bosco et al., 1982; Cutlip et al., 2004), the underlying mechanisms of the SSC‐effect are still controversially discussed (Seiberl et al., 2021; Seiberl, Power, & Hahn, 2015). Proposed mechanisms include energy return from the series elastic component of the muscle‐tendon unit (Cavagna & Citterio, 1974; Finni et al., 2001; Kawakami et al., 2002; Lindstedt et al., 2001), muscle stiffening and activation from stretch‐induced reflex responses (Dietz et al., 1979; Nichols & Houk, 1973; Taube et al., 2012), preactivation/preload of muscles due to activation dynamics (Bobbert et al., 1996; Cutlip et al., 2004; Fukutani et al., 2015; Fukutani et al., 2016; van Schenau et al., 1997; Walshe et al., 1998), and adaptive history‐dependent properties of muscle for example, by contribution of non‐cross‐bridge structures like titin (Fukutani et al., 2017; Seiberl, Power, Herzog, & Hahn, 2015; Tomalka et al., 2021). While it is not assumed that one of these mechanisms solely explains the SSC‐effect, the interaction of mechanisms is still not well understood (Seiberl et al., 2021).

Generally, it is complicated, maybe even impossible, to estimate the 1‐dimensional (1D) behavior of fascicle length changes in a 3D muscle based on joint movement alone. This is due to the decoupling of the fascicle behavior from the length changes of the MTU and can be expressed by the ratio of MTU shortening velocity to the fascicle shortening velocity. This decoupling of MTU and fascicle velocity, also referred to as MTU gearing (Wakeling et al., 2011), architectural gearing (Azizi & Roberts, 2014) or simply gearing (Dick & Wakeling, 2017), occurs mainly due to two reasons: First, tendon compliance and second, muscle belly gearing of pennate muscles.

The compliant, viscoelastic tendon, which is in series to the muscle belly, decouples the muscle belly from MTU behavior and was referred to as tendon gearing (Wakeling et al., 2011). For example, during fixed‐end contractions (i.e., no joint movement) the tendon allows the muscle belly to shorten and lengthen while the MTU maintains its length. During dynamic contractions, the tendon and muscle belly can experience length changes in equal or opposite direction during MTU motion. As a result, the length changes and force potential of the in‐series muscle can be counterintuitive and significantly affected by tendon compliance (Roberts et al., 1997; Wakeling et al., 2011). As a result, muscle fascicles do not necessarily undergo SSCs during movements that are typically associated with MTU SSCs. For instance, it has been demonstrated that during running, the muscle fibers of the plantar flexors constantly shorten while the MTU undergoes a stretch‐shortening cycle (SSC) (Lichtwark et al., 2007). Similar behavior was found during single leg jumping tasks where again; no active muscle lenghening was detected during MTU stretch (Aeles & Vanwanseele, 2019). However, other studies identified an active fascicle stretch during similar movements in different muscles involved in those activities, such as countermovement jumps (Hoffman et al., 2022), walking (Rubenson et al., 2012), hopping (Dick et al., 2021), or stepping tasks (Werkhausen et al., 2017; Werkhausen et al., 2019). Other studies have indicated that in shortening contractions of preloaded muscles, tendon compliance results in muscle fascicle shortening at much lower speeds, when compared to non‐preloaded contractions (Holzer et al., 2023). Tendon gearing is particularly relevant for MTUs with relatively long tendons (Mörl et al., 2016), such as the triceps surae (Arnold et al., 2013; Bohm et al., 2019; Farris & Sawicki, 2012; Tennler et al., 2022).

The term belly gearing (Wakeling et al., 2011), also known as architectural muscle gearing (Azizi et al., 2008), refers to differences in muscle belly and fascicle velocities due pennation angle, fascicle rotation (i.e., changes in pennation angle) and muscle shape changes during muscle contraction. Belly gearing occurs in pennate muscles and has been shown to decrease the amount of fascicle shortening needed for a given amount of muscle belly shortening (Azizi et al., 2008; Wakeling et al., 2011). As a result, pennate muscles typically operate with a relative velocity advantage as the length changes of fascicles are amplified by the fascicle rotation, thereby increasing the shortening velocity of the muscle belly. The effect of pennation angle and fascicle rotation can be characterized as the muscle's gear ratio (muscle belly shortening velocity/fascicle shortening velocity) (Azizi et al., 2008; Azizi & Roberts, 2014; Wakeling et al., 2011). Further, the muscle's belly gearing ratio was found to be influenced by force (Dick & Wakeling, 2017; Randhawa et al., 2013). For instance, muscles operate at low gear during high force contractions (Eng et al., 2018), that is, fascicle rotation is reduced because the tendon compliance is decreased at high MTU forces. In contrast, during low force contractions muscles operate at a high gear with substantial fascicle rotation. Thus, despite contradicting opinions about the impact of pennation itself (Lieber, 2022), belly gearing is believed to have a major impact on a muscle's mechanical performance. In vivo it is challenging to determine 3D muscle shape changes during muscle action, and simplified 2D MTU models and 2D ultrasound measurements are typically used for estimation. However, 2D shape changes during muscle contractions, such as muscle belly thickness, width, and length, have previously been investigated in vivo (Dick & Wakeling, 2017; Maganaris et al., 1998; Randhawa et al., 2013).

The significance of this observation in the context of the SSC‐effect warrants attention. As previously pointed out, both the belly and tendon gearing mechanisms are sensitive to the magnitude of forces exerted on the MTU. During SSCs, the so‐called stretch‐induced transient force enhancement (tFE) leads to significantly increased forces at the onset of shortening when compared with preloaded fixed‐end shortening contractions (Pinniger et al., 2006; Weidner et al., 2022). Due to tendon compliance, these increased MTU forces at the onset of shortening due to tFE should result in a longer tendon and shorter muscle belly/fascicles, leading to a reduced fascicle shortening velocity during SSC shortening when compared with pure shortening contractions due to tendon compliance effects. In addition, the increased MTU forces during SSC shortening might result in favorable belly gearing effects regarding force production capacity (Dick & Wakeling, 2017; Randhawa et al., 2013). Further, Azizi and Roberts (Azizi & Roberts, 2014) showed that belly gearing minimize the stretch applied directly to fascicles as the MTU lengthens during eccentric contractions in pennate muscles. Such gearing effects might also be relevant for SSC shortening. As a result, both belly and tendon gearing potentially reduce fascicle shortening velocity during SSC shortening when compared with pure shortening contractions, subsequently leading to an increased muscle force production capacity according to the force‐velocity relationship (Hill, 1938; Holzer et al., 2023), which might help to explain the SSC‐effect. Despite the indications of MTU decoupling during SSC contractions, it has not previously been investigated in the context of the SSC‐effect.

In addition to MTU decoupling mechanisms, long lasting residual force enhancement (rFE) effects (Abbott & Aubert, 1952) might help to explain the SSC‐effect. However, following active muscle shortening, isometric steady‐state forces are typically decreased compared to the corresponding purely fixed‐end contractions: a property referred to as residual force depression (rFD) (Chen et al., 2019; Edman, 1976; Lee & Herzog, 2003). Therefore, in a context of SSCs, the long‐lasting history dependence effects manifest as reduced rFD rather than rFE.

Besides other factors (Bakenecker et al., 2020, 2022), tFE has been shown to be greater during fast contractions (Weidner et al., 2022) and in in vitro studies rFE was shown to increase with stretch amplitude (Weidner et al., 2022). However, this is not necessarily the case for in vivo contractions (Bakenecker et al., 2022; Hahn et al., 2007; Seiberl, Power, Herzog, & Hahn, 2015). Therefore, fast contractions with large stretch amplitudes might show greater tFE and rFE. As demonstrated, larger tFE should potentially influence MTU decoupling due to tendon compliance. Even thought, belly gearing was found to be affected by force but not velocity during shortening (Dick & Wakeling, 2017; Randhawa et al., 2013) the velocity dependent starting force in SSC shortening according to the eccentric force‐velocity relationship, leads to velocity effects on the belly gearing ratio. Therefore, both stretch amplitude and stretch velocity potentially influence the SSC‐effect.

The aim of this study was to examine the interplay between force enhancement and MTU decoupling during in vivo SSC shortening and how this interplay affects the SSC‐effect. According to previous studies, we expected lower fascicle velocities during SSC shortening when compared with pure shortening (SHO) contractions due to a force‐related decoupling within the MTU. Thereby, we differentiate between tendon and belly gearing as possible contributors to the SSC‐effect, which has previously not been accounted for in this field of research. To test this, SHO, SSC, and fixed‐end reference contractions were elicited by electrical nerve stimulation to minimize the contribution of potential reflex responses and to ensure a constant muscle activation throughout all contraction conditions, which is hard to achieve voluntarily. As belly gearing has been shown to vary with force level, and due to the force‐length relationship, we tested a large and small range of motion (ROM) of the ankle joint, with stretch phases in SSCs ending at different joint angles. In addition, we implemented contraction velocity as a third factor, as stretch‐induced tFE is velocity dependent and might therefore trigger MTU decoupling mechanisms differently in slow and fast SSCs.

## 2 METHODS

### 2.1 Participants

Sixteen participants provided written informed consent prior to participating in the study. Exclusion criteria included any musculoskeletal or neurological impairments in the right lower limb. After a familiarization session, five participants (three male, two female) were not invited to return for the measurement session due to the inability to obtain analyzable ultrasound images (*n* = 2), failure to achieve reproducible stimulation of the tibial nerve (*n* = 2), or difficulty fitting two ultrasound probes on the calf due to limited space (*n* = 2). The remaining eleven male participants (age: 30 ± 5 years, height: 179 ± 5 cm, body mass: 79 ± 7 kg) returned on a separate day to complete the measurement session. The experimental protocol was approved by the Faculty of Sport Science Ethics Committee at Ruhr University Bochum and conducted according to the main principles of the Declaration of Helsinki.

### 2.2 Experimental setup

**FIGURE 1:**
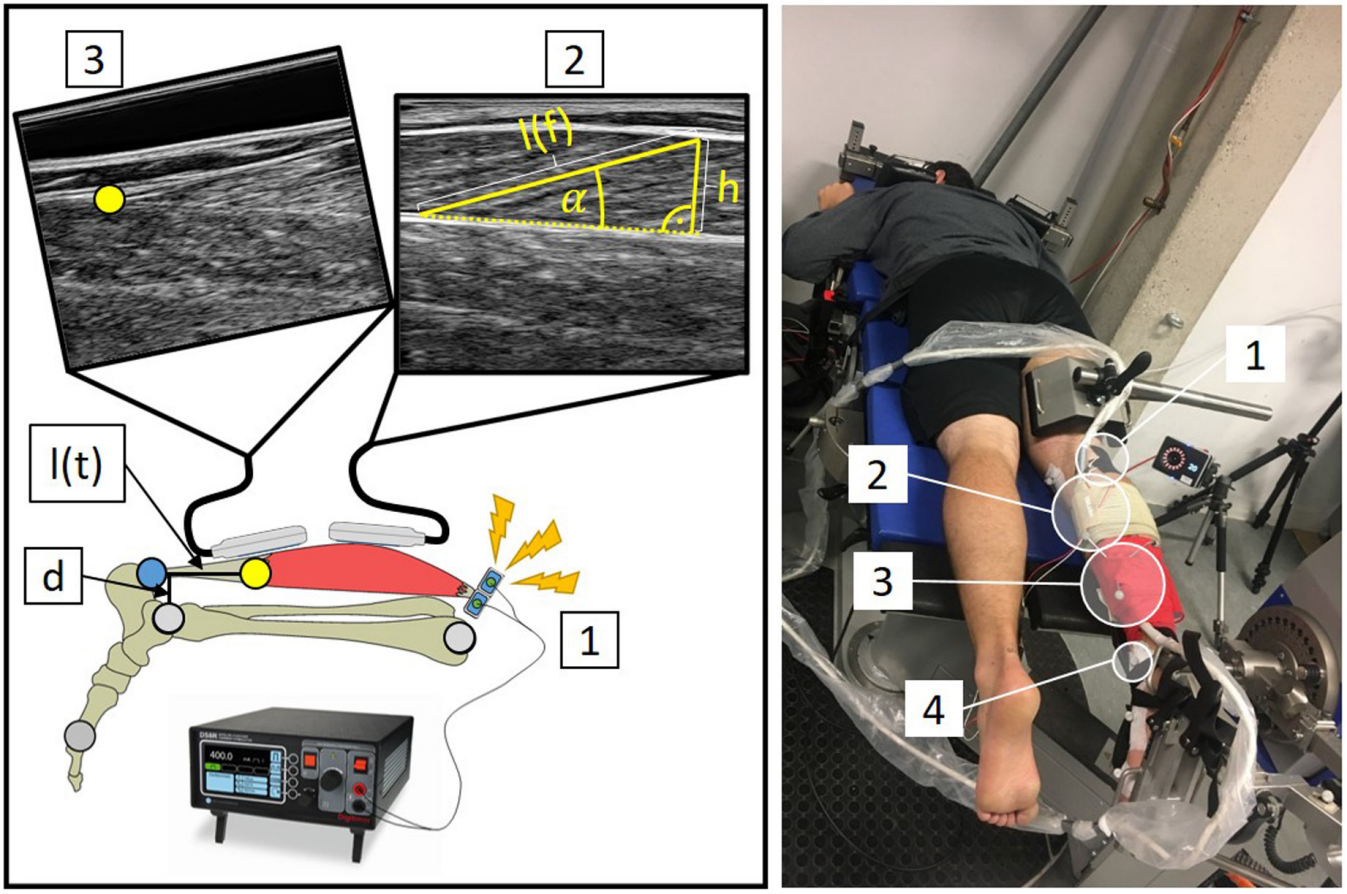
Experimental setup: Participants lay prone on the bench of a dynamometer with their shoulders, hip, thigh and foot tightly secured. (1) Electrical stimulation of the tibialis nerve. (2) Ultrasound placed on the gastrocnemius medialis muscle belly to determine fascicle length (l(f)) and pennation angle (α). (3) Ultrasound placed on the myotendinous junction (MTJ) to track the proximal end of the Achilles tendon. (4) Reflective markers for 3D motion capture of leg kinematics and ultrasound probe position. Achilles tendon length (l(f)) was defined as the linear distance from MTJ to its distal insertion point marked by a virtual marker. Minimal distance of the Achilles tendon to the joint axis was defined as the Achilles tendon moment arm (d). Muscle thickness (h) was calculated trigonometrically using fascicle length and pennation angle measurements.

Net joint torque and ankle joint kinematics during electrically stimulated right‐legged plantar flexion contractions were measured using a motor‐driven dynamometer (IsoMed2000, D&R Ferstl, GmbH, Hemau, GER) and a 3D motion capture system (Vicon Peak, Oxford, UK) operating at 1000 and 200 Hz, respectively. Two 60 mm linear ultrasound probes (Echo Blaster 128 CEXT LV7.5/60/128Z‐2, UAB Telemed, Lithuania) operating at 61.5 Hz were used to assess fascicle length change and myotendinous junction (MTJ) displacement of the gastrocnemius medialis (GM) during contractions (Figure 1). Throughout the measurement, participants lay in a prone position with their knee and hip joints fully extended (Figure 1). The right foot was tightly strapped to a customized foot plate, attached to the dynamometer lever, to minimize heel displacement. The rotational axis of the dynamometer was visually aligned with the ankle's estimated axis of rotation at rest.

### 2.3 Electrical stimulation

For electrical nerve stimulation of the tibial nerve, a constant current stimulator (DS7AH, Digitimer, UK) was used. The self‐adhesive anode electrode was placed in the distal area of the popliteal fossa. At the beginning of the measurement session, single electrical square‐wave pulses with 20 mA were used to find the individual optimal cathode position (highest plantar flexion torque response) using a hand‐held cathode pen within the proximal area of the popliteal fossa. Once the position was determined, the cathode electrode was fixed to this site using additional tape for reducing the pain experienced during electrical stimulation (Cattagni et al., 2018). Involuntary muscle activation was induced using tetanic electrical stimulation (50 Hz, square‐wave pulses with 1 ms pulse width). During short stimulated plantar flexion contractions (<2 s) at 15° dorsiflexion (DF, 0° refers to tibia axis perpendicular to the plantar aspect of the foot) the stimulation intensity (i.e., the current) was slowly increased until a steady plantar flexion torque of 60% of the participants' maximum voluntary plantar flexion contraction (MVC) net joint torque was reached. The maximum plantar flexion torque for the corresponding ankle joint angle was previously determined during three MVC contractions.

### 2.4 Experimental protocol

All participants attended one familiarization session prior to the measurements to get used to the test situation and procedures. Further, this session was used to determine whether the volunteering participants were eligible for the actual experiments due to reasons listed in the participants section.

In the measurement session, each participant performed a standardized warm‐up to precondition the MTU (Maganaris, 2003). Three MVCs were then performed at 15° DF (plantar flexion: PF), which served as a reference for setting the stimulation intensity for the nerve stimulation. Participants were verbally encouraged during all MVCs.

**FIGURE 2:**
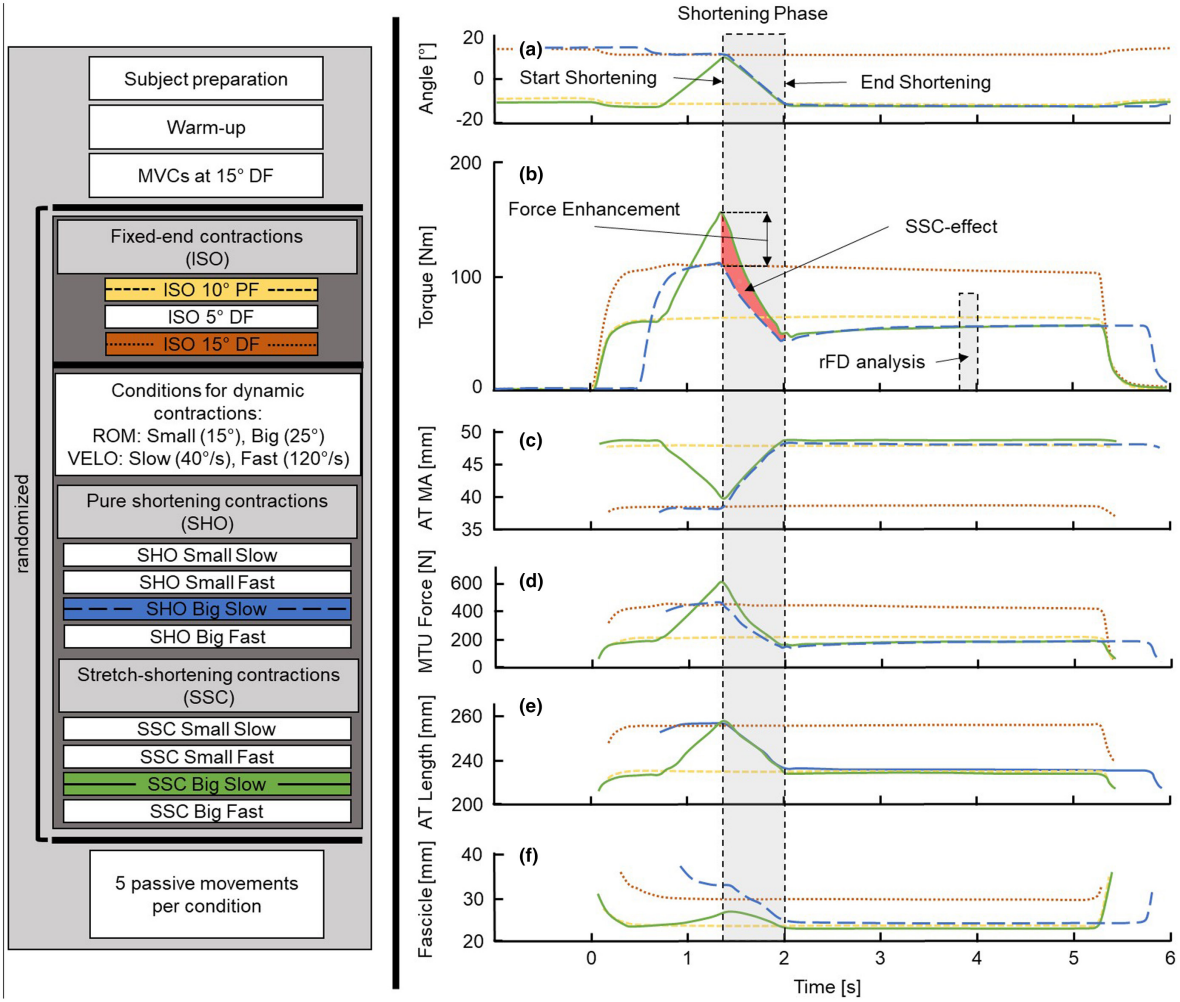
Experimental protocol and corresponding exemplar data of one set of conditions of one participant. Protocol (left): After subject preparation and standardized warm‐up, maximum voluntary contractions were assessed at 15° dorsiflexion. The following 11 submaximal (60% MVC) plantar flexion contractions were elicited by electrical nerve stimulation. The protocol included fixed‐end reference contractions (ISO) in plantar and dorsi flexed positions (PF and DF, respectively), pure shortening (SHO) and stretch‐shortening cycles (SSC) at FAST (120°/s) and SLOW (40°/s) dynamometer crank arm velocities (VELO) each with a LARGE (25°) and SMALL (15°) range of motion (ROM) of the ankle joint in a randomized order. Afterwards, passive rotations were recorded for all conditions. Exemplary data (right): Sagittal joint kinematics (a), torque (b), dynamic Achilles tendon (AT) moment arm (c), gastrocnemius medialis muscle‐tendon unit (MTU) force (d), AT length (e), and fascicle length (f) of one set of conditions of one participant. Colors refer to the contraction conditions highlighted in the protocol flowchart (left). Data analysis was focused on the shortening phase (gray shaded area). Stretch‐induced transient torque/force enhancement was defined as the ratio of the eccentric force/torque just prior to shortening to the time‐matched corresponding fixed‐end contraction. Increased toques/forces during the shortening phase represent the SSC‐effect. Residual force depression (rFD) was analyzed as the mean forces/torques of the SSC and SHO trials 1.8–2 s after the end of shortening relative to the corresponding ISO contractions.

Eleven different submaximal contractions were elicited for each participant including fixed‐end (ISO) and dynamic contractions. Dynamic contractions included the contraction conditions (CONTYPE) pure shortening (SHO) and stretch‐shortening contractions (SSC). All dynamic trials were performed over different ROMs (*LARGE* = 25°, between 10°PF and 15°DF; *SMALL* = 15°, between 10°PF and 5°DF) and dynamometer crank arm velocities (VELO) (*FAST* = 120°/s; *SLOW* = 40°/s). ISO trials were recorded at the target positions of the dynamic trials (i.e., 10°PF, 5°DF and 15°DF). All submaximal trials were recorded in a randomized order. Each tetanic contraction lasted 5 s (Figure 2). Rotation of the dynamometer was automatically triggered 0.5 s after plantar flexion torque reached 95% of the REF force potential at the starting ankle joint angle, ensuring a fixed‐end holding phase prior to any dynamometer rotation (Figure 1). The dynamometer crank arm was accelerated at 400°/s^2^. Trials were repeated when (a) fixed‐end torque prior to rotation was >10% higher than the REF torque determined for the same ankle joint angle; (b) rotation was not initiated within the first 2 s of the contraction; or (c) the shape of the stimulated torque‐time curve was found to be abnormal by the principal investigator (e.g., no steady torque development during fixed‐end phases before and after rotation). On average 1 ± 1 out of the 11 submaximal trials had to be repeated within a measurement.

After the active contractions, participants had to remain strapped to the dynamometer for passive joint rotations (relaxed muscle) at the aforementioned dynamometer kinematics. Each passive rotation was recorded five times. Again, trials were repeated if the torque‐time curve was found to be abnormal (i.e., indicating an unintentional contraction of the plantar flexor muscles) by the principal investigator.

### 2.5 Kinematics

Ankle joint kinematics of the right foot were analyzed by the 10‐camera Vicon motion analysis system synchronized with the dynamometer (Figure 1). Reflective markers (14 mm) on predefined anatomical landmarks defined the ankle joint. The anatomical landmarks were: lateral malleolus, medial malleolus, lateral femoral condyle, and medial femoral condyle (most medial/lateral aspect respectively), first and fifth metatarsophalangeal joint, and navicular (most medial aspect). Data were captured using the Vicon Nexus software (Oxford Metrics, Oxford, UK) and filtered using a 6 Hz fourth order (zero lag) low pass filter. Data was then transferred into MATLAB (The MathWorks, Inc., Natick, Massachusetts, United States) for further analysis. Joint angles were defined relative to the segment angles measured in the standardized static reference position (knee fully extended, shank longitudinal axis perpendicular to the sole of the foot). Kinematic data was up sampled to 1000 Hz using spline interpolation. All reported angles refer to ankle flexion and extension in the sagittal plane. For statistical analysis, we analyzed individually calculated ankle joint ROM during the shortening phase.

### 2.6 Muscle‐tendon behavior

GM fascicle length and pennation angle were assessed using one of the ultrasound probes (7 MHz; image depth 50 mm), carefully placed at ~50% of GM length over the mid‐longitudinal axis. Another identical ultrasound probe was placed on the GM myotendinous junction (MTJ) in order to track its displacement. Both transducers were secured to the leg in a custom‐made mount using self‐adhesive tape and bandages (Figure 1). Three reflective markers were rigidly attached to the mount of the MTJ ultrasound probe. A voltage signal generated by the ultrasound systems was used to synchronize all devices. Fascicle length and pennation angle (defined as the linear distance between a fascicle's insertion to the superficial and deep aponeuroses and as the angle between fascicles and the deep aponeurosis, respectively) was manually determined throughout all trials using the “segmented line” tool in ImageJ (ImageJ v.1.48; National Institutes of Health, USA). The location of the MTJ was manually tracked using the “straight line” tool in ImageJ. All tracking was done by four experienced investigators. Each participant was tracked by only one investigator who was then cross‐checked by one of the other investigators. Ultrasound data was filtered using a 10 Hz fourth order (zero lag) low pass filter and upsampled to 1000 Hz using spline interpolation (MATLAB). By using the image coordinate system and previously obtained calibration of the transducer coordinate system, the position of the MTJ was reconstructed into the three dimensional coordinate system of the laboratory (Lichtwark & Wilson, 2005; Werkhausen et al., 2019). GM muscle length was defined as the linear distance between the medial femoral condyle and MTJ (Figure 1). GM free Achilles tendon (AT) length was defined as the linear distance between MTJ and the osteotendinous junction on the calcaneus which was previously identified using ultrasound and palpation and tracked as a virtual marker in reference to reflective markers placed on the foot segment (Figure 1). The shortest perpendicular distance between the estimated AT force vector and the ankle joint axis was defined as the AT moment arm at each time point (Figure 1) (Manal et al., 2013; Obst et al., 2017; Werkhausen et al., 2019). This dynamic AT moment arm is sensitive to changes due to joint rotation and contraction intensity (Figure 2) (Holzer et al., 2020). GM muscle thickness ($h_{\mathrm{GM}}$) was calculated using fascicle length ($l_{f}$) and pennation angle: $h_{\mathrm{GM}}=l_{f}*\sin\left(\alpha_{\text{pennation angle}}\right)$. The projected belly length ($l_{b}$) was taken as the length of the fascicle projected onto the lower aponeurosis and calculated as $l_{b}=l_{f}*\cos\left(\alpha_{\text{pennation angle}}\right)$. GM belly gearing ratio ($G_{b}$) was calculated as the ratio of projected belly shortening velocity ($v_{b}$) and fascicle shortening velocity ($v_{f}$) ($G_{b}=v_{b}/v_{f}$). For statistical analysis, we calculated the length change and mean velocity of the GM muscle fascicles, AT tendon and MTU during the shortening phase. Further, change of GM belly gearing ratio, GM pennation angle, projected belly length, and GM muscle thickness during the shortening phase was analyzed.

### 2.7 Kinetics

All torque data was smoothed using a 10 Hz fourth order (zero lag) low pass filter and corrected for gravitational and passive forces due to a stretching of the MTU and the moment of inertia during dynamometer acceleration. For this, the torque‐time series during the five passive trials was averaged and subtracted from the corresponding stimulation trials. AT force was estimated from the net ankle joint torque divided by the individual dynamic AT moment arm (Figure 2). GM MTU force was estimated by weighting the calculated AT force according to the anatomical proportion of the plantar flexors (15.9% based on the physiological cross‐sectional area; (Fukunaga et al., 1996)). Joint/MTU work was calculated by the cumulated angular joint rotation/MTU length change multiplied by ankle joint torque/GM force along the AT during the shortening phase, respectively. Fascicle work were calculated as the cumulated fascicle length change multiplied by the calculated fascicle force ($F_{\text{fascicle}}=F_{\text{along tendon}}/\cos\left(\alpha_{\text{pennation angle}}\right)$) during the shortening phase. The SSC‐effect was calculated for all structural levels as the ratio between joint, MTU and fascicle work performed during SSC shortening and the work performed during shortening in the corresponding SHO trials (Figure 2). For the analysis of residual force depression (rFD) mean force during a 0.2 s time period at 1.8–2 s after the end of shortening were used for statistical analysis, and was compared to the corresponding isometric reference forces (Figure 2). Stretch‐induced force enhancement was defined as the ratio between the eccentric force just prior to SSC shortening and the corresponding time‐matched fixed‐end REF contraction force (Figure 2).

### 2.8 Statistics

Normality of distribution and homogeneity of variance were checked by the Shapiro–Wilk and Levene's test (*p* > 0.05). A three‐way repeated measure analysis of variance (rmANOVA) was performed to identify significant differences in various dependent variables (e.g., shortening work, fascicle length change, …) during active muscle shortening across different conditions (independent variables: VELO, ROM and CONTYPE (SSC vs. SHO)). A two‐way rmANOVA was performed to identify changes in the SSC‐effect (work ratio SSC to SHO) across different conditions (independent variables: VELO and ROM; dependent variable: SSC‐effect). In case sphericity assumptions were violated, the Greenhouse–Geisser correction was used. With significant main effects, post‐hoc paired *t*‐tests were used to test for significant differences within the different CONTYPEs. Thereby, a Bonferroni–Holm correction was used to adjust the α‐levels. Repeated‐measures correlation (Bakdash & Marusich, 2017) were implemented to examine the relationship between torque/force enhancement just prior to the shortening phase and the SSC‐effect using the web application provided by Marusich et al. (Marusich & Bakdash, 2021). Statistical parametric mapping paired *t*‐tests (SPM) were performed using MATLAB (The MathWorks, Inc., Natick, Massachusetts, United States), comparing calculated MTU force of SSC and SHO trials during the shortening phase using open‐source package spm1d (www.spm1d.org, 55, (Pataky et al., 2013)). All other statistical tests were performed using the computer software JASP (JASP Team, 2023. JASP Version 0.16.1) with the level of significance set at *α* = 0.05. All data in text and figures are presented as mean ± standard deviation.

## 3 RESULTS

The results section presents all the statistical main results important for the discussion. The entire set of parameters and outcomes of the rmANOVAs is presented in the supplementary material—Data S1.

At shortening onset, we found significant tFE with torques enhanced by 24% ± 12% in SSC contractions (*p* < 0.001 [*η* ^2^ = 0.73]). Stretch‐induced torque enhancement was positively affected by FAST VELO (*p* = 0.043 [*η* ^2^ = 0.006]) with no effect of ROM. Statistical parametric mapping analysis showed that torques and MTU forces remained enhanced throughout SSC shortening (each *p* < 0.001).

The SSC‐effect was confirmed for mean joint torque, MTU force and fascicle force (joint: 19% ± 12% [*p* < 0.001, *η* ^2^ = 0.58], MTU: 22% ± 14% [*p* < 0.001, *η* ^2^ = 0.43], fascicle: 22% ± 17% [*p* < 0.001, *η* ^2^ = 0.37]). This effect was more prominent in FAST contractions as shown by the significant interaction of VELO and CONTYPE on all structural levels (joint: *p* = 0.004 [*η* ^2^ = 0.04], MTU: *p* = 0.007 [*η* ^2^ = 0.03], fascicle: *p* = 0.017 [*η* ^2^ = 0.02]). The comparison of mean joint torque and mean MTU and fascicle force ratios (SSC/SHO) confirmed this increased SSC‐effect for FAST contractions when compared with SLOW (joint: 8% ± 9% [*p* < 0.004, *η* ^2^ = 0.29], MTU: 9% ± 9% [*p* < 0.001, *η* ^2^ = 0.31], fascicle: 9% ± 1%, [*p* = 0.006, *η* ^2^ = 0.27]).

**FIGURE 3:**
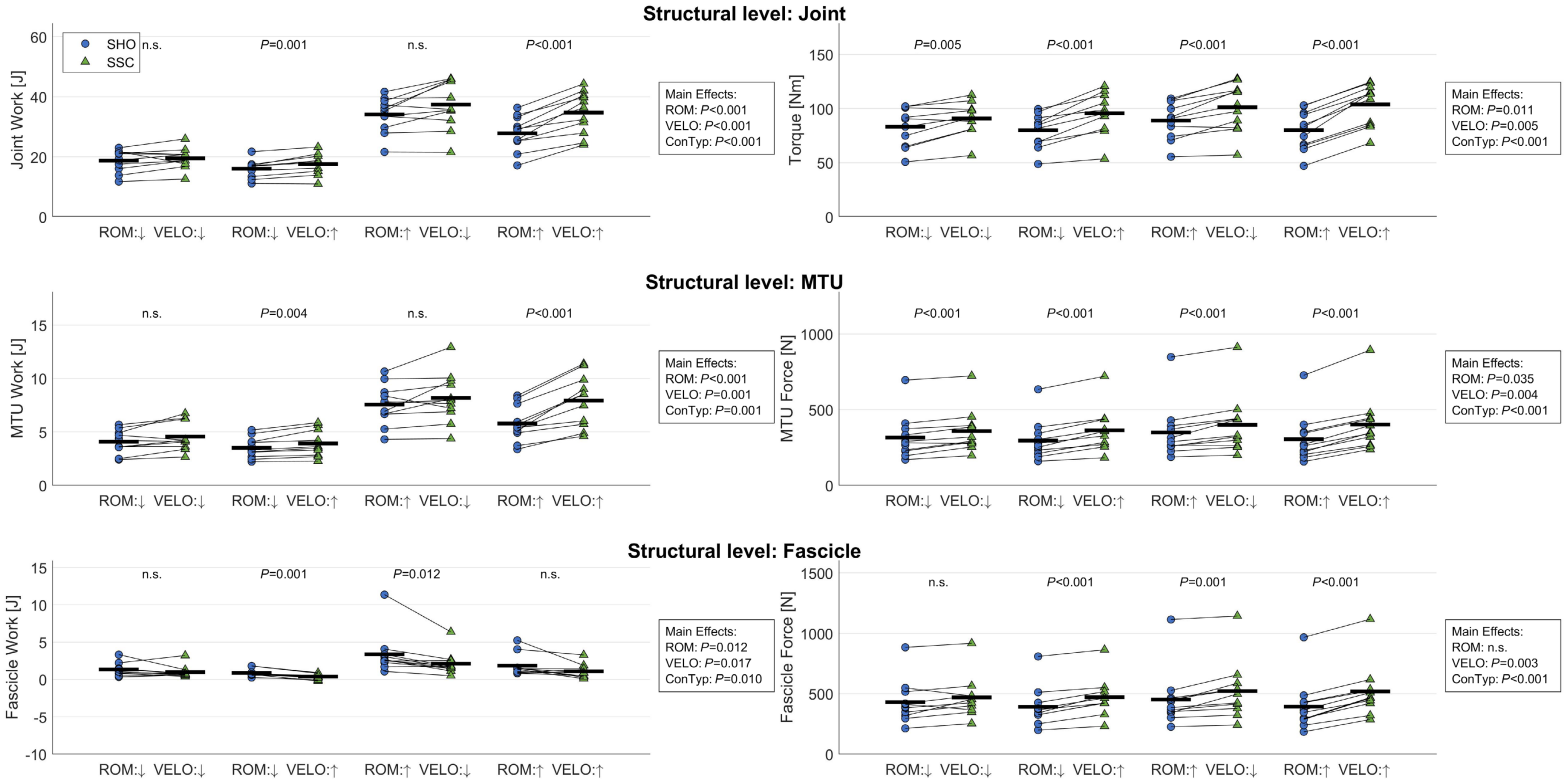
Mean (−) and subject‐specific (o) work during shortening for the ankle joint, gastrocnemius medialis muscle‐tendon unit (MTU) and muscle fascicle for contraction conditions pure shortening (SHO) and stretch‐shortening contractions (SSC) at SMALL (↓) and LARGE (↑) range of motion (ROM), and SLOW (↓) and FAST (↑) dynamometer velocity (VELO). Three‐way rmANOVA revealed significant main effects of ROM, VELO and contraction type (ConTyp) (*p* < 0.05). *p*‐values in the graph indicate a significant difference (*p* < 0.05) between SHO and SSC at the same ROM and VELO in case of a significant ConTyp effect.

The SSC‐effect was also confirmed for mean joint and MTU work (Figure 3); joint: 12% ± 13% [*p* < 0.001, *η* ^2^ = 0.83], MTU: 17% ± 18% [*p* < 0.001, *η* ^2^ = 0.74]. This effect was more prominent in FAST contractions as shown by the significant interaction of VELO and CONTYPE on all structural levels (joint: *p* = 0.012 [*η* ^2^ = 0.01], MTU: *p* = 0.015 [*η* ^2^ = 0.01]). The comparison of work ratios (SSC/SHO) confirmed this increased SSC‐effect for FAST contractions when compared with SLOW (joint: 5% ± 10% [*p* = 0.007, *η* ^2^ = 0.11], MTU: 9% ± 9% [*p* = 0.013, *η* ^2^ = 0.12]). In contrast, MG fascicle work was reduced by 35% ± 41% (*p* < 0.001 [*η* ^2^ = 0.24]) during SSC shortening (Figure 3) with no VELO effect on the work ratios (fascicle: *p* = 0.073 [*η* ^2^ = 0.14]).

**FIGURE 4:**
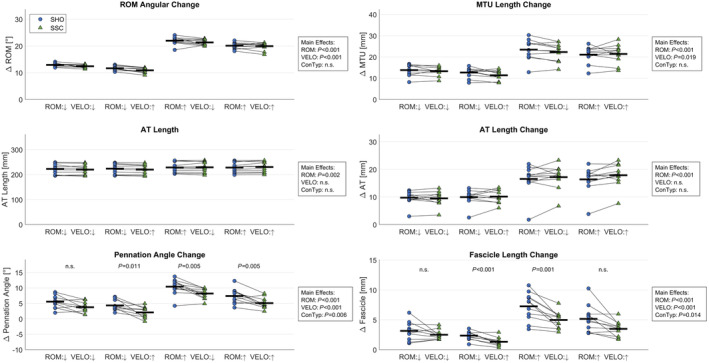
Mean (−) and subject‐specific (o) kinematics during shortening for the ankle joint, gastrocnemius medialis muscle‐tendon unit (MTU) and muscle fascicle for contraction conditions pure shortening (SHO) and stretch‐shortening contractions (SSC) at SMALL (↓) and LARGE (↑) range of motion (ROM), and SLOW (↓) and FAST (↑) dynamometer velocity (VELO). Achilles tendon (AT) length refers to its length at the onset of shortening. Three‐way rmANOVA revealed significant main effects of ROM, VELO and contraction type (ConTyp) (*p* < 0.05). *p*‐values in the graph indicate a significant difference (*p* < 0.05) between SHO and SSC at the same ROM and VELO in case of a significant ConTyp effect.

ROM and MTU length change during shortening was unaffected by the CONTYPE (Figure 4; *p* = 0.053 [*η* ^2^ < 0.01] and *p* = 0.221 [*η* ^2^ < 0.01], respectively). Yet, we found significant differences between SSC and SHO shortening phases regarding fascicle behavior. Fascicle length change and shortening velocity were significantly reduced during SSC shortening compared with SHO (Figure 4; −58% ± 72% [*p* = 0.009, *η* ^2^ = 0.13], −32% ± 46% [*p* = 0.039, *η* ^2^ = 0.07], respectively). GM pennation angle change was significantly reduced during SSC shortening compared with SHO (Figure 4; −44% ± 12% [*p* = 0.006, *η* ^2^ = 0.14]). Projected muscle length change was significantly reduced during SSC shortening compared with SHO (−31% ± 29% [*p* = 0.009, *η* ^2^ = 0.13]). The resulting GM belly gearing ratio (mean = 1.34) was unaffected by CONTYPE (*p* = 0.212, [*η* ^2^ = 0.05]).

GM AT length at shortening onset, AT shortening velocity, and AT length change throughout the shortening phase were unaffected by the CONTYPE (Figure 4; *p* = 0.457 [*η* ^2^ = 0.01], *p* = 0.131 [*η* ^2^ < 0.01], and *p* = 0.127 [*η* ^2^ < 0.01], respectively). Muscle thickness at shortening onset was unaffected by the CONTYPE (*p* = 0.140 [*η* ^2^ = 0.05]).

There were no differences regarding the rFD of 10% ± 8% between SSC and SHO (*p* = 0.399 [*η* ^2^ < 0.01]). Residual force depression was unaffected by VELO (*p* = 0.847 [*η* ^2^ < 0.01]). Greater ROM led to increased residual force depression (SMALL: 7.3%, LARGE: 12.5%, *p* = 0.034 [*η* ^2^ = 0.19]).

## 4 DISCUSSION

The aim of this study was to investigate the interplay between force enhancement and MTU decoupling mechanisms during in vivo SSC contractions and how it affects the SSC‐effect. Different angular velocities and ROMs were compared to better understand the biomechanical mechanisms underpinning the SSC‐effect, precisely the increased force and shortening work in SSCs compared with SHO conditions. Our data showed clear SSC‐effects on all structural levels. In contrast to the increased shortening fascicle forces in SSCs during shortening, fascicle work was reduced during SSCs when compared to SHO resulting in a previously unreported “reversed SSC‐effect.” We found significantly reduced fascicle shortening velocities and pennation angle changes during the shortening phase between both conditions, which led to an increased force production capacity of the fascicles during SSC cycles compared with SHO. This effect was more prominent during fast contraction velocities and seems to play an important role for the SSC‐effect.

In the current study, the SSC‐effect was confirmed for torque, MTU force and fascicle force, which were significantly increased under SSC compared with SHO conditions. Furthermore, shortening work was increased at the joint and MTU level, however, not at the fascicle level (Figure 3). The increased work at the joint and MTU levels are a result of the increased torques and forces, as ROM and MTU length changes were unaffected by contraction type. The increase of torque and MTU force during SSCs at the onset of shortening can be attributed to tFE (Weidner et al., 2022). The increased starting torque seems to play an important role in the explanation of the SSC‐effect as shown by the significant positive repeated measures correlation found between tFE and the SSC‐effect (*p* < 0.001, *r* = 0.67). However, torque and MTU force were increased throughout the entire shortening phase in all conditions as confirmed by the statistical parametric mapping analysis. Therefore, tFE might not explain the enhanced forces during SSC shortening by itself.

We found a reduced fascicle length change during SSCs compared with SHOs ultimately leading to slower shortening velocities during SSCs compared with SHO. According to the force‐velocity relationship (Hill, 1938), slower shortening velocity is favorable regarding the fascicle's force production capacity and thus likely explains the increased torques/forces observed throughout the entire shortening phase. The ratio between mean fascicle contraction velocity during SSC and SHO was unaffected by VELO (*p* = 0.329) and ROM (*p* = 0.754). Therefore, we believe that MTU decoupling occurs similarly regardless of angular velocity or ROM. The reduced shortening velocity might be the result of the increased force level at the onset of the shortening. In theory, larger forces pulling on the tendon should lead to an increased tendon length and a reduced muscle belly length and hence, most likely a reduced fascicle length as previously described by Wakeling et al., (2011). However, this was not the case in our data as AT length at the onset of shortening was not different between SSC and SHO (*p* = 0.457) despite a mean difference in force of 124 ± 53 N (ranging from 82 ± 44 to 160 ± 34 N depending on ROM and VELO). Chang et al. (2020) reported stiffness values between ~500 N/mm for the GM Achilles sub tendon during plantar flexion contractions in a similar setup with a comparable participant group. According to their data, the expected length difference in the AT due to stretch‐induced tFE would therefore only be around 0.25 mm (ranging from 0.16 to 0.32 mm depending on ROM and VELO). These expected length differences are likely within the ultrasound measurement error that needs to be considered when interpreting our results concerning AT shortening velocity and AT length that did not differ during the shortening phase and therefore would be unaffected by the stretch (*p* = 0.203, *p* = 0.127, respectively).

The decreased fascicle length change during SSC shortening was accompanied by a reduced fascicle angle change which resulted in a reduced projected belly length change of 1.9 ± 1.8 mm during SSC shortening compared with SHO shortening. As a result, GM belly gearing ratio was unaffected by CONTYPE. Further, we did not detect any differences in muscle thickness between SSC and SHO that might accompany potential differences regarding belly gearing. Therefore, our data does not show clear evidence that belly gearing is a contributor to the SSC‐effect. Although, we did show clear signs of beneficial MTU decoupling resulting in an increased muscle force potential that benefits the SSC‐effect, our data cannot explain the reason behind the reduced fascicle shortening velocities. However, it should be mentioned that in a typical 2D MTU model, the reduced changes in fascicle length, fascicle angle, and projected belly length during shortening can only be explained with an increased Achilles tendon length at SSC shortening onset. As there is not yet a technical solution to measure 3D dynamic muscle architecture accurately, 2D ultrasound measurements in future studies should even more focus on more sensitivity to reliably detect small length differences in the Achilles tendon. This could include high‐speed ultrasound with a better spatio‐temporal resolution (Hauraix et al., 2015) or taking Achilles tendon curvature into account (Kharazi et al., 2021). However, it should be noted that we identified a significantly increased AT length at the onset of shortening in the LARGE ROM conditions when compared with SMALL ROM which was expected due to the force‐length relationship (Holzer et al., 2020). Therefore, the method used for AT length measurements seems to be sensitive to detect length changes once force differences are large enough.

In contrast to our findings, Fukutani et al. (2019) reported that fascicle length change in the shortening phase was unaffected by CONTYPE. However, it is important to note that their ultrasound data is based on single frames at a relatively low frame rate of 30 Hz, and no image progression was analyzed. Further, studies investigating MTU behavior during locomotion and jumping tasks concluded that the compliance of the tendon allows the muscle fascicles to shorten at a slower speed during SSCs (Bohm et al., 2019, 2021; Finni et al., 2001; Kawakami et al., 2002; Lichtwark et al., 2007). Thereby, it is important to note that these conclusions are not based on ultrasound data of tendon length but of the muscle belly. Therefore, belly gearing effects might have been partially misattributed as tendon gearing effects.

Our data indicate that the SSC‐effect in the triceps surae is more affected by changes in contraction velocity than by changes in ROM. The statistical analysis of the work and force ratios clearly show that faster contractions lead to an increased SSC‐effect. This might be attributed to MTU decoupling effects as significant interactions and post‐hoc analysis revealed that the effect of a reduced fascicle shortening velocity is more prominent in fast contractions.

Energy return form the series elastic component of the muscle‐tendon unit has been proposed as a major contributor to the enhanced forces during the shortening phase of SSCs compared with SHO (Cavagna & Citterio, 1974; Lindstedt et al., 2001). In the current study, AT tendon shortening velocity and AT length change throughout the shortening phase were unaffected by the preceding stretch (*p* = 0.203 and *p* = 0.127, respectively). Thus, even though it makes sense that the increased forces at the end of the stretch led to an increased AT tendon length, where energy is stored and later returned during shortening, we were not able to detect this.

Besides the changed muscle‐tendon interaction and increased initial forces due to the mechanisms of tFE, long‐lasting history dependence effects could also be a contributor to the observed SSC‐effect. It is argued that the mechanisms of residual force enhancement are triggered during stretch and counteract the mechanisms of rFD in the shortening phase (Seiberl, Power, Herzog, & Hahn, 2015). In in vivo experiments this involvement of rFE effects is typically indirectly interpreted from reduced rFD during the steady‐state isometric phase following shortening (Groeber et al., 2020; Seiberl, Power, Herzog, & Hahn, 2015). However, we could not find significant differences between the residual force depression observed following SSC compared with SHO conditions. This is in line with results from Fortuna et al. (2018) where neither the peak force at the end of stretch, nor the work performed during shortening affected residual force depression following a SSC. However, other in vivo experiments in humans have shown clearly reduced rFD (Fortuna et al., 2018; Hahn & Riedel, 2018) or even increased forces (Seiberl, Power, Herzog, & Hahn, 2015) as a consequence of active stretch prior to shortening. The cause of this in vivo variability in muscle history dependence effects is not clear and subject of current investigation (Jacob et al., 2023; Paternoster et al., 2021). Some of these differences are likely related to preload related initial force depression (Raiteri & Hahn, 2019), muscle length and type and intensity of activation. For example, in a study by Groeber et al. (2020), in contrast to submaximal muscle action, only MVCs of the m. quadriceps femoris led to less depressed forces post shortening after SSC contractions when compared with pure shortening. Further, all contractions in the current study ended at relatively short muscle lengths on the ascending limb of the force‐length relation (Holzer et al., 2020) where residual force enhancement is unlikely to occur (Bakenecker et al., 2020). Accordingly, although not identified in the steady‐state phase after shortening, we still found stretch induced force enhancement and cannot exclude that residual force enhancement contributed to the increased MTU forces by counteracting residual force depression effects during the shortening phase of SSCs. However, there is strong data from different groups proofing the SSC‐effect in isolated muscles and even in skinned fibers (Fukutani et al., 2019; Fukutani & Herzog, 2021; Tomalka et al., 2020) where sarcomeric mechanisms leave the only reasonable explanation.

Due to the difficulties of assessing muscle force and contraction velocity directly, muscle kinematics and kinetics are typically derived from angular velocities and external force measurement devices or inverse dynamic approaches, respectively. The underlying assumption that joint performance also reflects on the performance of the MTU could be problematic due to an incorrect estimation of muscle‐tendon force and due to possible co‐contractions of antagonistic and agonistic muscles, unaccounted for in the overall net joint torque. Yet, our results show that a direct transfer of joint work seems like a decent approach to estimate the work done by the entire MTU when analyzing movements with identical joint ROM. This assumption was confirmed to be reasonable in our very controlled setting, as the calculated shortening work measured on a joint level behaved similar to the estimated triceps surae MTU work (Figure 3). Thereby, MTU/joint work was increased during SSC contractions when compared with SHO contractions, which is in line with a large number of experiments on various structures (in vitro, in situ, in vivo) (Fukutani et al., 2017; Groeber et al., 2021; Hahn & Riedel, 2018; Seiberl, Power, Herzog, & Hahn, 2015; Tomalka et al., 2020).

In contrast to that, we were surprised by the extent of which MTU decoupling seems to limit the transferability of joint and MTU work to fascicle work. Fascicle work during SSC shortening was reduced when compared with SHO contractions (Figure 3). This is in line with studies investigating MTU behavior during locomotion/jump tasks where tendon compliance led to muscle fascicles shortening at much lower speeds, optimizing output and efficiency (Aeles et al., 2018; Bohm et al., 2019, 2021; Finni et al., 2001; Kawakami et al., 2002; Lichtwark et al., 2007). This highlights the complex interaction of contractile and elastic components of the MTU. In SSCs, the MG MTU seems to increase joint and MTU work, while fascicle work is reduced.

### 4.1 Limitations

In this study, it was assumed that GM constantly contributes 15.9% of the total plantar flexion moment. However, this is an over simplistic model, as the remaining muscles of the triceps surae (gastrocnemius lateralis, soleus), in addition to the six additional ankle plantar flexors that are not attached onto the AT (plantaris, tibialis posterior, flexor hallucis longus, flexor digitorum longus, peroneus brevis, and peroneus longus), might show differences in MTU interaction and SSC‐effect contribution.

A further aspect that needs to be discussed as a limitation is that our data is based on male subjects only, as participants needed to fulfill the requirements to take part in the current study (e.g., enough space on shank for two US probes; see methods section). Previous investigations have demonstrated sex differences in mechanical properties of the triceps surae muscle‐tendon unit, indicating that females tend to generate less muscle and tendon forces, exhibit shorter tendon lengths and smaller cross‐sectional areas, and demonstrate more compliant tendons with a lower Youngs's modulus compared with their male counterparts (Burgess et al., 2009; Deng et al., 2021; Kubo et al., 2003; Lepley et al., 2018; Tennler et al., 2022; Zhang et al., 2021). Therefore, the observed MTU decoupling effect is likely to be influenced by sex. However, the main finding of the impact of MTU to fascicle decoupling on the SSC‐effect, and the compromised transferability of angular work measurements on the joint level to fascicle work, should hold true regardless of potential sex differences in MTU properties.

Finally, in this study we measured fascicle and tendon behavior of the GM using 2D ultrasound. Therefore, all data and interpretations are based on the assumption that a single fascicle in one plane represents the behavior of the entire muscle which is obviously flawed. However, tracking of 3D muscle dynamics during dynamic contractions is currently impossible. In addition, we did not track the behavior of the aponeurosis which potentially changes its properties depending on the contraction condition (Böl et al., 2015; Eng & Roberts, 2018; Raiteri et al., 2018). However, this is currently also not possible during dynamic in vivo muscle contractions and only possible for isolated muscle specimens. During the MTU stretch, work might be stored as elastic energy within the aponeurosis (Azizi & Roberts, 2009; Roberts et al., 1997; Siebert et al., 2012) and is later returned during the shortening. Further research should investigate the contribution of the aponeurosis on the SSC‐effect.

## 5 CONCLUSION

This study shows that the SSC‐effect is, among other contributors, the result of a changed muscle fascicle force production capacity due a reduced fascicle shortening velocity. Usually, the SSC‐effect is defined by increased force and increased work. However, we clearly showed that this is not necessarily the case for in vivo experiments where fascicle force was increased whereas fascicle work was reduced during SSC shortening when compared to SHO. However, the reduced fascicle work is not a “reversed SSC‐effect” but reflects the efficiency of MTU SSCs with performance enhancements by increasing external joint torque, work and power, while reducing muscle fascicle work.

Our findings also show that experiments on the SSC‐effect conducted on isolated muscles and muscle fibers might not transfer directly to in vivo experiments where the complex MTU structure affects important variables such as fascicle shortening velocity that cannot be controlled. This must be considered in future data interpretations.

## AUTHOR CONTRIBUTIONS

Denis Holzer, Daniel Hahn, Tobias Sieber, Wolfgang Seiberl conception and design of research; Denis Holzer performed the experiments; Denis Holzer analyzed data; Denis Holzer and Wolfgang Seiberl interpreted results of experiments; Denis Holzer prepared figures; Denis Holzer and Wolfgang Seiberl drafted the manuscript; Denis Holzer, Daniel Hahn, Tobias Sieber., Wolfgang Seiberl edited and revised manuscript; Denis Holzer, Daniel Hahn, Tobias Sieber, Ansgar Schwirtz, Wolfgang Seiberl approved final version of manuscript.

## FUNDING INFORMATION

This project was funded by the Deutsche Forschungsgemeinschaft (DFG, German Research Foundation) under Grants SI 841/15‐1,2, HA 5977/5‐1,2, and SE 2109/2‐1,2; project number 354863464.

## CONFLICT OF INTEREST STATEMENT

The authors declare no competing interests.

## Supporting information


Data S1.

Table S1.

## Data Availability

The datasets generated during and/or analyzed during the current study are available from the corresponding author on reasonable request.
