# Supplementary material for: Decoupling of muscle‐tendon unit and fascicle velocity contributes to the in vivo stretch‐shortening cycle effect in the male human triceps surae muscle
Source: Physiol Rep. 2024 Dec 11;12(23):e70131. doi: 10.14814/phy2.70131 (PMC11634487; doi:10.14814/phy2.70131)
Supplement: Supplementary file 1 — Data S1. [file PHY2-12-e70131-s002.docx]

| Denis Holzer  Technical University of Munich  Faculty of Sport and Health Sciences \| Associate Professorship of Biomechanics in Sports  Uptown München – Campus D  Georg-Brauchle-Ring 62 \| 80992 Munich \| Germany  Tel.: +49 89 289 24587  Fax: +49 89 289 24582  Mail: denis.holzer@tum.de  www.sg.tum.de/biomechanik |
| --- |

Munich, 03. September 2024

Dear Editors,

This letter accompanies the submission of our revised manuscript entitled “Decoupling of muscle-tendon unit and fascicle velocity contributes to the in vivo stretch-shortening cycle effect in the human triceps surae muscle“.

The way muscle force is generated and transferred to the skeletal system has been intensively researched to better understand how muscle-tendon interaction benefits human and animal locomotion. During the most common muscle action in locomotion, the stretch-shortening cycle (SSC), shortening muscle force and work have been shown to be increased when compared to pure shortening contractions. A variety of underlying mechanisms behind this so called “SSC-effect” have been discussed. While it is not assumed that one of these mechanisms solely explains the SSC-effect, the interaction of mechanisms is still not well understood.

In our manuscript we show that the *in vivo* SSC-effect is, among other contributors, the result of a changed muscle fascicle force production capacity due to a reduced fascicle shortening velocity. For the first time we were able to demonstrate that the decoupling of the muscle-tendon unit and muscle fascicles is a contributor to the SSC-effect. Further, we show that for *in vivo* experiments fascicle force can be increased whereas fascicle work is reduced during SSC shortening when compared to pure shortening contractions. This reflects the efficiency of muscle-tendon unit SSCs resulting in performance enhancements by increasing external joint torque, work and power, while reducing muscle fascicle work.

We consider Physiological Reports to be a good platform for our research, as our biomechanical approach is covered by the aims and scope of Physiological Reports. Our findings are of interest for many researchers in our field, e.g. the previously published work of Fortuna et al. (2017, 2019)* or Seiberl et al. (2015)* investigating the stretch-shortening cycle on in vivo muscle-tendon unit behavior.

The manuscript is not under consideration elsewhere and will not be submitted for publication elsewhere until a final decision is made by you as to the acceptability of the manuscript. All authors were fully involved in the study and preparation of the manuscript. The research represents original work without fabrication, fraud or plagiarism and we, the authors, take full responsibility for the content of the manuscript. We hope that you find the manuscript and its topic of great interest and we thank you for your consideration.

Yours sincerely,

D. Holzer, D. Hahn, A. Schwirtz, T. Siebert, W. Seiberl

*Fortuna et al., 2017: <https://doi.org/10.14814/phy2.13279>

*Fortuna et al., 2019: <https://doi.org/10.14814/phy2.14188>

*Seiberl et al., 2015: <https://doi.org/10.14814/phy2.12401>
